# Long non-coding RNA *HOTAIR* is associated with human cervical cancer progression

**DOI:** 10.3892/ijo.2014.2758

**Published:** 2014-11-17

**Authors:** HEE JUNG KIM, DAE WOO LEE, GA WON YIM, EUN JI NAM, SUNGHOON KIM, SANG WUN KIM, YOUNG TAE KIM

**Affiliations:** 1 Institute of Women’s Life Medical Science, Division of Gynecologic Oncology, Department of Obstetrics and Gynecology, Yonsei University College of Medicine, Seoul, Republic of Korea; 2 Department of Obstetrics and Gynecology, Yonsei University Graduate School, Seoul, Republic of Korea; 3 Department of Obstetrics and Gynecology, Bucheon St. Mary’s Hospital, Catholic University College of Medicine, Bucheon, Republic of Korea

**Keywords:** *HOTAIR*, invasion, metastasis, prognosis, cervical cancer

## Abstract

The functions of many long non-coding RNAs (lncRNAs) in human cancers remain to be clarified. The lncRNA Hox transcript antisense intergenic RNA (*HOTAIR*) has been reported to reprogram chromatin organization and promote breast and colorectal cancer metastasis, the involvement of lncRNAs in cervical cancer is just beginning to be studied. In the present study, we examined the expression and the functional role of *HOTAIR* in cervical cancer. *HOTAIR* expression was determined in cervical cancer tissues (n=111) and corresponding normal tissues (n=40) by using real-time polymerase chain reaction, and its correlation with clinical parameters and prognosis were analyzed. To determine the effect of *HOTAIR* knockdown and overexpression in cervical cancer cell lines, we used the CCK-8 assay, wound healing migration and Matrigel invasion assay. The expression level of *HOTAIR* in cervical cancer tissues was higher than that in corresponding non-cancerous tissues. High *HOTAIR* expression correlated with lymph node metastasis, and reduced overall survival. A multivariate analysis showed that *HOTAIR* was a prognostic factor for predicting cervical cancer recurrence. Knockdown of *HOTAIR* reduced cell proliferation, migration, and invasion in cervical cancer cell lines. Moreover, *HOTAIR* regulated the expression of vascular endothelial growth factor, matrix metalloproteinase-9 and epithelial-to-mesenchymal transition (EMT)-related genes, which are important for cell motility and metastasis. Therefore, *HOTAIR* may promote tumor aggressiveness through the upregulation of VEGF and MMP-9 and EMT-related genes. These findings indicate that *HOTAIR* may represent a novel biomarker for predicting recurrence and prognosis and serve as a promising therapeutic target in cervical cancer.

## Introduction

Non-coding RNAs (ncRNAs) are found in the genome of humans, mouse and other animals. However, the functions of ncRNAs are only partially understood. ncRNAs are mainly classified into housekeeping or regulatory ncRNAs (1–3). Based on transcript size, regulatory ncRNAs can be further grouped into 2 subclasses: small ncRNAs (20–200 nt) and long ncRNAs (lncRNAs, >200 nt). microRNAs (miRNAs) have been the most extensively investigated of the small ncRNAs, and estimates suggest that >1,000 miRNAs regulate up to 30% of all protein-encoding genes (4–7). Characterization of the functional and clinical significance of some ncRNAs has shown that they are key factors in gene regulation and influence normal and cancer cell phenotypes (4,8–10).

Recent data have demonstrated that >3,000 human long intervening non-coding RNAs (lincRNAs) and most long ncRNAs are associated with DNA-binding proteins such as chromatin-modifying complexes (11) and epigenetically regulate the expression of multiple genes (12,13). Transcription of lncRNAs has been shown to modulate gene activity in response to external oncogenic stimuli and DNA damage (14). This finding indicates the potential involvement of lncRNAs in the pathogenesis of human diseases, most notably in cancer (15). HOX transcript antisense intergenic RNA (*HOTAIR*) is a 2158-bp lncRNA that was identified from a custom tiling array of the *HOXC* gene cluster. Interaction of *HOTAIR* with the polycomb repressive complex 2 (PRC2), which is composed of EZH2, SUZ12 and EED, leads to the trimethylation of histone H3 lysine 27 and establishment of the repressive H3K27me3 chromatin mark (11). *HOTAIR* has been shown to inhibit tumor suppressor genes such as *HOXD10*, *PGR*, and the proto-cadherin gene family in breast cancer cells (16). *HOTAIR* is a negative prognostic factor for breast, liver, colon, pancreatic and cervical cancer (17–19). Furthermore, increased *HOTAIR* expression has been correlated with enhanced breast and colon cancer metastasis. Although *HOTAIR* has been shown to play a critical role in the progression of breast, liver, colon and pancreatic cancers, little is known about the molecular mechanisms in cervical cancer.

Cervical cancer is the third most common cancer and the fourth leading cause of cancer death in women worldwide (20). Widespread implementation of Pap smear screening programs in recent years has decreased the incidence and mortality of cervical cancer in many countries (21). Despite this, cervical cancer continues to be a major public health problem (21). Cancer cell motility and invasion play a crucial role in the mortality of cervical cancer patients (22). Therefore, major research efforts have focused on the identification of tumor-specific markers for predicting the biological behavior of cervical cancers. Several miRNAs, including miR-214, miR-143, miR-375, miR-23b and miR-20, have been shown to modulate cervical cancer cell motility and invasion; these may represent potential prognostic markers for predicting the aggressiveness of cervical cancer (23–27). Increased understanding of the molecular mechanisms underlying cervical carcinogenesis and progression is required to identify reliable prognostic markers associated with tumor aggressiveness.

In the present study, we determined the expression and clinical significance of *HOTAIR* in cervical cancer. We found that *HOTAIR* was highly expressed in cervical cancer and was associated with disease recurrence. Furthermore, *HOTAIR* knockdown inhibited proliferation, migration and invasion of human cervical cancer cell lines. Also, we examined the molecular events that occur downstream of *HOTAIR* involvement in cervical cancer migration and invasion. These findings provide novel insights into the role of *HOTAIR* in the metastatic progression of cervical cancer.

## Materials and methods

### Human tissues

Cervical cancer samples were obtained from 111 female patients who underwent surgery at Yonsei Severance Hospital, Yonsei University, between 2007 and 2012. Specimens from patients with newly diagnosed invasive [FIGO (International Federation of Gynecology and Obstetrics) stage IA-IVB] cervical cancer who had not received prior treatment were included in the study. Forty samples of normal cervix from patients undergoing simple hysterectomy because of uterine leiomyomata were obtained as controls. Specimens from patients with concomitant gynecological cancer were excluded from the study. All specimens were immediately frozen in liquid nitrogen and stored at −80°C until RNA extraction. The study was conducted according to the principles in the Declaration of Helsinki and was approved by the ethical committee of Yonsei Severance Hospital. Informed consent was obtained from all patients. The clinical information is summarized in Table I.

### Cell culture

SiHa (squamous cervical carcinoma), HeLa (epitheloid cervical carcinoma) and Caski (epidermoid cervical carcinoma established from a metastasis in the small bowel mesentery) human cervical cancer cell lines obtained from the American Type Culture Collection (ATCC, Rockville, MD, USA). SiHa and HeLa cells were cultured in Dulbecco’s modified Eagle’s medium, and Caski cells were cultured in RPMI-1640 medium (Gibco-BRL, Gaithersburg, MD, USA). The human keratinocyte cell line HaCaT was cultured in RPMI-1640 medium. The culture media were supplemented with 10% (vol/vol) fetal bovine serum and penicillin/streptomycin. The cell lines were maintained at 37°C in a humidified atmosphere of 5% CO_2_ and 95% air. Cells with a passage number <20 were used in all experiments.

### Quantitative real-time polymerase chain reaction (qRT-PCR)

Total RNA was extracted from cancerous/non-cancerous specimens or cell lines using TRIzol^®^ reagent (Invitrogen, Carlsbad, CA, USA), and 2 μg of total RNA was reverse transcribed into first-strand cDNA by using a reverse transcription reagent kit (Invitrogen) according to the manufacturer’s protocol. qRT-PCR was performed using the SYBR^®^ Green real-time PCR kit (Toyobo, Co., Ltd., Osaka, Japan) in a 20-μl reaction volume, which contained 10 μl of SYBR-Green Master PCR Mix, 5 pmole each of forward and reverse primers, 1 μl of diluted cDNA template, and appropriate amounts of sterile distilled water. Conditions for the amplification of genes were as follows: initial denaturation at 95°C for 3 min; 40 cycles of denaturation at 95°C for 15 sec, annealing at 60°C for 60 sec, and elongation at 72°C for 60 sec; and final elongation at 72°C for 5 min. qRT-PCR was performed on the ABI StepOnePlus Real-Time PCR system (Applied Biosystems, Foster City, CA, USA). All quantifications were performed with *U6* as the internal standard. The PCR primer sequences were as follows: *HOTAIR*, 5′-GGTAGAAAAAGCAACCACGAAGC-3′ (sense) and 5′-ACATAAACCTCTGTCTGTGAG TGCC-3′ (antisense); E-cadherin, 5′-ATTCTGATTCTGC TGCTCTTG-3′ (sense) and 5′-AGTAGTCATAGTCCTGGTCCT-3′ (antisense); β-catenin, 5′-TGCAGTTCGCCTTCACTATG-3′ (sense) and 5′-ACTAGTCGTGGAATGGCACC-3′ (antisense); vimentin, 5′-TGGATTCACTCCCTCTGGTT-3′ (sense) and 5′-GGTCATCGTGATGCTGAGAA-3′ (antisense); snail, 5′-GAGGCGGTGGCAGACTAG-3′ (sense) and 5′-GACACATCGGTCAGACCAG-3′ (antisense); twist, 5′-CGGGAGTCCGCAGTCTTA-3′ (sense) and 5′-TGAATCTTGCTCAGCTTGTC-3′ (antisense); and *U6*, 5′-CTCGCTTCGGCAGCACA-3′ (sense) and 5′-AACGCTTCAGGAATTTGCGT-3′ (antisense). Relative gene expression was analyzed using the 2^−ΔΔCT^ method, and the results were expressed as extent of change with respect to control values. qRT-PCR experiments were replicated at least 3 times.

### Small interfering RNA (siRNA) transfection

*HOTAIR* siRNA (siHOTAIR-1 and siHOTAIR-2) and negative control siRNA (siNC) were purchased from Bioneer (Daejeon, Korea). Cells (5×10^4^ cells/well) were seeded into 6-well plates and were transfected with 10 nM siRNA in phosphate-buffered saline (PBS) using the G-Fectin kit (Genolution Pharmaceuticals Inc., Seoul, Korea) according to the manufacturer’s protocol. These siRNA-transfected cells were used in the *in vitro* assays 48 h post-transfection. The target sequences for *HOTAIR* siRNAs were as follows: siRNA-1, 5′-UUUUCUACCAGGUCGGUAC-3′ and siRNA-2, 5′-AAUUCUUAAAUUGGGCUGG-3′.

### Plasmid constructs and the generation of stable cell line

The human *HOTAIR* transcript variant 3 cDNA was amplified by PCR and was inserted into the pLenti6/V5-D-TOPO vector according to ViraPower™ Lentiviral Expression systems (Invitrogen). Briefly, plasmid was transfected into the 293FT cell line and then lentivirus was infected in desired cell line. Selection of *HOTAIR* stable transfected cells was performed in medium containing blasticidin (Invitrogen).

### Cell proliferation assay

Cell proliferation was evaluated using the Cell Counting Kit-8 (CCK-8) assay (Dojindo Laboratories, Kumamoto, Japan). Cells (2×10^3^ cells/well) were seeded into 96-well flat-bottomed plates in 100 μl of complete medium. The cells were incubated overnight to allow for cell attachment and recovery and were then transfected with siNC or siHOTAIR for 24, 48, 72 and 96 h. CCK-8 solution (10 μl) was added to each well, and the cells were incubated for an additional 2 h. Absorbance was measured at 450 nm using a microplate reader. Three independent experiments were performed in triplicate.

### Matrigel invasion assay

The Matrigel invasion assay was performed using the BD BioCoat Matrigel Invasion Chamber (pore size: 8 mm, 24-well; BD Biosciences, Bedford, MA, USA) according to the manufacturer’s protocol. siHOTAIR-transfected cells and siNC-transfected cells (5×10^4^ cell/plate) were plated in the upper chamber in serum-free medium, and complete medium was added to the bottom chamber. The Matrigel invasion chamber was incubated for 48 h at 37°C under 5% CO_2_. Non-invading cells were removed from the upper chamber using cotton-tipped swabs. Cells that had invaded through the pores onto the lower side of the filter were stained (Diff-Quik; Sysmex, Kobe, Japan), and these were counted using a hemocytometer. The number of invaded siHOTAIR-transfected cells was expressed as fold-change relative to siNC-transfected cells, which was set at 1. The assay was replicated at least 3 times.

### Wound healing migration assay

Cells transfected with siNC or siHOTAIR (5×10^5^ cells/well) were seeded into 6-well culture plates with serum-containing medium and were cultured until the cell density reached ~90% confluence. The serum-containing medium was removed, and cells were serum starved for 24 h. When the cell density reached ~100% confluence, an artificial homogeneous wound was created by scratching the monolayer with a sterile 200-μl pipette tip. After scratching, the cells were washed with serum-free medium. Images of cells migrating into the wound were captured at 0, 24 and 48 h using a microscope. The assay was performed in triplicate.

### Western blot analysis

Cells were transfected with siNC or siHOTAIR for 48 h, washed with ice-cold 0.01 M PBS (pH 7.2), and lysed in lysis buffer [50 mM Tris-HCl (pH 7.4), 150 mM saline, 1% Nonidet P-40, and 0.1% sodium dodecyl sulfate (SDS)] supplemented with protease inhibitors. Protein concentrations were determined using Bio-Rad protein assay reagent according to the Bradford method (Bio-Rad Laboratories, Hercules, CA, USA). Samples were boiled for 5 min, subjected to 10% SDS-PAGE, and transferred electrophoretically to polyvinylidene difluoride membranes (Millipore, Billerica, MA, USA). Membranes were blocked with 5% non-fat dried milk in 1X Tris-buffered saline containing 0.1% Tween-20 (TBST; pH 7.6) at room temperature for 1 h and were then incubated with primary antibody at 4°C overnight under constant agitation. The primary antibodies used included: rabbit anti-human VEGF (1:500 dilution; Abcam, Cambridge, MA, USA), rabbit anti-human MMP-9 (1:1,000 dilution; Cell Signaling Technology, Beverly, MA, USA), rabbit anti-human E-cadherin (1:1,000 dilution; Cell Signaling Technology), rabbit anti-human β-catenin (1:1,000 dilution; Cell Signaling Technology), mouse anti-human Vimentin (1:1,000 dilution; Sigma, St. Louis, MO, USA), mouse anti-human Snail (1:1,000 dilution; Cell Signaling Technology), rabbit anti-human Twist (1:1,000 dilution; Abcam), and mouse anti-human β-actin antibody (1:5,000 dilution; Sigma). Membranes were washed 3 times with 1X TBST, incubated with a horseradish peroxidase-conjugated anti-rabbit secondary antibody (1:2,000 dilution; Abcam) or anti-mouse secondary antibody (1:2,000 dilution; Abcam) for 1 h at room temperature under constant agitation, and then washed 3 times with 1X TBST. Proteins were visualized using an enhanced chemiluminescence system (ECL™; Amersham, Little Chalfont, UK), and band intensities were quantified using the Luminescent image analyzer (LAS 4000 mini; Fujifilm, Uppsala, Sweden).

### Statistical analysis

SPSS software (standard version 20.0; IBM) was used for all statistical analyses. Data are expressed as the mean ± standard deviation (SD). The association between *HOTAIR* expression and clinicopathological characteristics was assessed using the Pearson’s χ^2^ test, Student’s t-test, and Fisher’s exact test. Overall survival was analyzed by the Kaplan-Meier method, and the differences between groups were estimated by the log-rank test. Multivariate survival analysis was performed for the significant parameters in the univariate analysis using the stepwise Cox regression model analysis. All statistical tests were two-sided, and P<0.05 was considered to indicate a statistically significant result.

## Results

### Association between HOTAIR expression and clinicopathologic factors in cervical cancer

**Figure 1:**
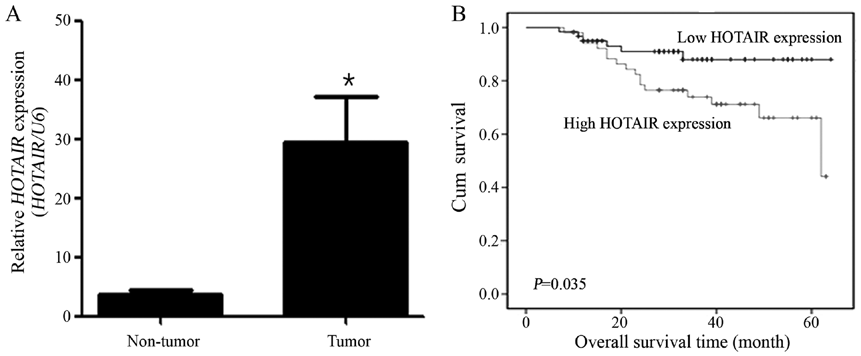
Relative *HOTAIR* expression and its clinical significance. (A) *HOTAIR* expression was significantly higher in cervical cancer tissues (n=111) than in non-cancerous tissues (n=40). Relative *HOTAIR* expression was determined using qRT-PCR with *U6* as an internal control. Data are expressed as mean ± SD. ^*^P<0.05 vs. non-tumor control. (B) Kaplan-Meier overall survival curves of the patients with cervical cancer and different levels of *HOTAIR* (log-rank test; P=0.035).

The expression of *HOTAIR* lncRNA was determined in cervical cancer tissues (n=111) and corresponding normal tissues (n=40) using qRT-PCR. *HOTAIR* expression in cervical cancer tissues was >30-fold that in non-cancerous tissues (Fig. 1A), suggesting that the expression of *HOTAIR* is upregulated in cervical cancer. To evaluate the prognostic value of *HOTAIR* for predicting clinical outcome in cervical cancer, *HOTAIR* expression levels were determined in an independent panel consisting of 111 cervical cancer patients with extensive clinical follow-up (Table I). The patients were divided into low (n=22) and high (n=89) *HOTAIR* expression groups, and clinicopathologic features were compared between the two groups. Age, stage, cell type and lymphatic invasion were not significantly different between the low and high *HOTAIR* expression groups. In contrast, *HOTAIR* expression was correlated with lymph node metastasis (P=0.0437). Multivariate Cox regression model analysis was performed to further evaluate the prognostic significance of *HOTAIR* expression and clinicopathologic characteristics on recurrence (Table II). *HOTAIR* expression was a significant prognostic indicator for recurrence in cervical cancer patients (relative risk=5.281; P=0.0493). As shown in Fig. 1B, *HOTAIR* expression levels were correlated with overall survival *HOTAIR* (log-rank test; P=0.035). These data suggest that *HOTAIR* expression represent an independent prognostic factor for survival and that the overexpression of *HOTAIR* might play an important role in the program of cervical cancer.

### HOTAIR knockdown decreases cell proliferation in cervical cancer cells

**Figure 2:**
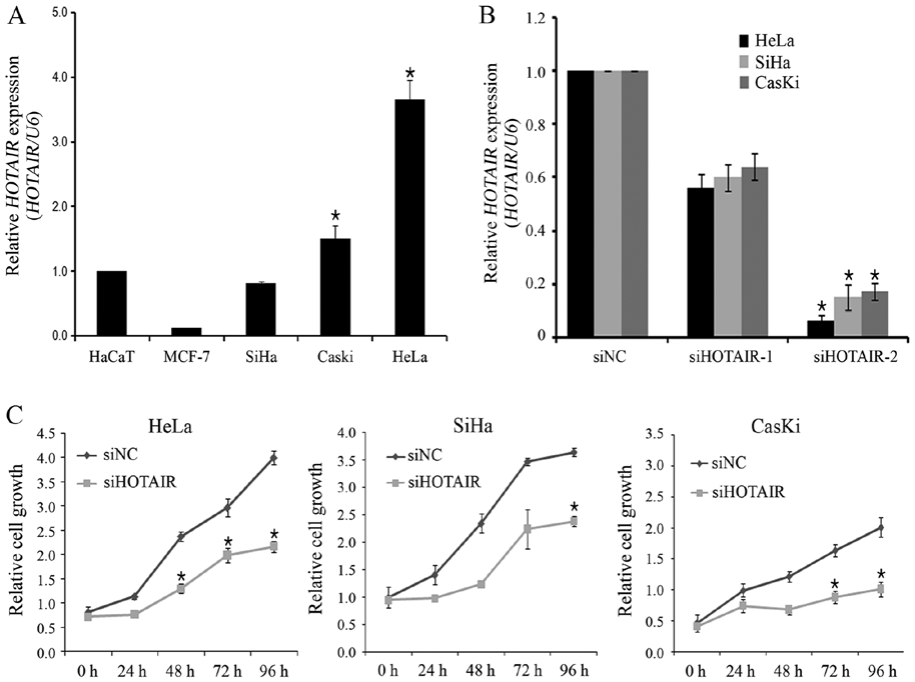
Knockdown of *HOTAIR* inhibits the cell proliferation of cervical cancer cells. (A) Expression of *HOTAIR* in cervical cancer cells. *HOTAIR* expression was evaluated using qRT-PCR with *U6* as an internal control. (B) Cells were transfected with *HOTAIR*-specific siRNA and negative control siRNA (siNC), and knockdown efficiency was determined by qRT-PCR analysis. (C) Knockdown of *HOTAIR* decreases cell proliferation in HeLa, SiHa and CasKi cells. The proliferation of cervical cancer cells transfected with siHOTAIR and negative control siRNA (siNC) was determined using the CCK-8 assay. Bars indicate mean ± SD of three independent experiments performed in triplicate. ^*^P<0.05 vs. siNC.

To determine the functional role of *HOTAIR* in cervical cancer, siRNA was used to downregulate *HOTAIR* expression. For this, *HOTAIR* expression in SiHa, Caski and HeLa cervical cancer cell lines was first determined using qRT-PCR. As shown in Fig. 2A, *HOTAIR* expression levels were higher in HeLa cells than in SiHa and Caski cells. Therefore, HeLa cells were used for siRNA-mediated knockdown of *HOTAIR* expression. The knockdown efficiency of the 2 *HOTAIR*-specific siRNAs (siHOTAIR-1 and siHOTAIR-2) was evaluated, and siHOTAIR-2 was found to have higher silencing efficiency than siHOTAIR-1 did (Fig. 2B). Therefore, siHOTAIR-2 was selected for use in the subsequent *in vitro* biological assays. To determine the role of *HOTAIR* in cervical cancer cell growth, siHOTAIR-transfected cells were used in the CCK-8 assay. siRNA-mediated knockdown of *HOTAIR* decreased cell proliferation by 30% at 96 h post-transfection in HeLa cells (Fig. 2C). Also, *HOTAIR* siRNA inhibited cell proliferation in SiHa and Caski cells. This finding indicates that *HOTAIR* is involved in the proliferation of cervical cancer cells.

### HOTAIR promotes cervical cancer cell migration and invasion

**Figure 3:**
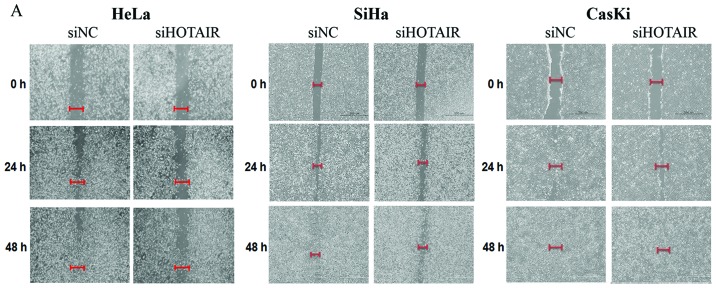

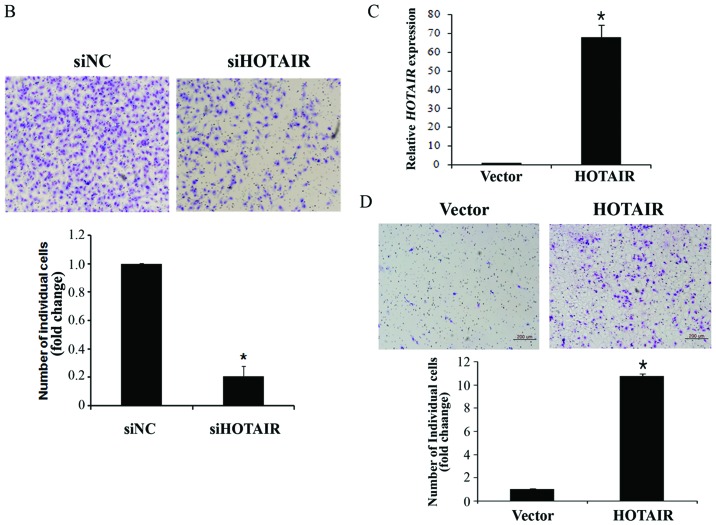
*HOTAIR* knockdown inhibits the migration and invasion of cervical cancer cells. (A) Wound healing assay was used to determine migration in siHOTAIR-transfected HeLa SiHa and CasKi cells (magnification, ×200). (B) Matrigel invasion assay was used to determine invasion after 48 h in HeLa cells. (C) Overexpression of *HOTAIR* in SiHa cells analyzed by qRT-PCR. (D) Cell invasion was evaluated using Matrigel invasion chamber. Overexpression of *HOTAIR* in SiHa cells increased the invasive capacity after 48 h. Each assay was performed in triplicate. Data are mean ± SD. ^*^P<0.05 vs. siNC.

To investigate the effect of *HOTAIR* on migration and invasion, siHOTAIR-transfected cells were used in wound healing and Matrigel invasion assays, respectively. The width of the wound closure was larger in siHOTAIR-transfected cells than in siNC-transfected of HeLa, SiHa and Caski cells (Fig. 3A). Therefore, downregulation of *HOTAIR* decreased the migration of cervical cancer cells. We also tested whether *HOTAIR* knockdown has an inhibitory effect on HeLa cell invasion. Knockdown of *HOTAIR* inhibited HeLa cell invasion >80% (Fig. 3B). To further assess the role of *HOTAIR* in the pathogenesis of cervical cancer, SiHa cell lines stably expressing ectopic *HOTAIR* were established (Fig. 3C). Consistent with the previous results, stable *HOTAIR* overexpression in SiHa cells resulted in a significantly increase the invasion ability of SiHa cells (Fig. 3D). Collectively, these results indicate that *HOTAIR* has an important role in the migratory and invasive phenotype of cervical cancer cells.

### HOTAIR upregulates VEGF and MMP-9 expression in cervical cancer cells

**Figure 4:**
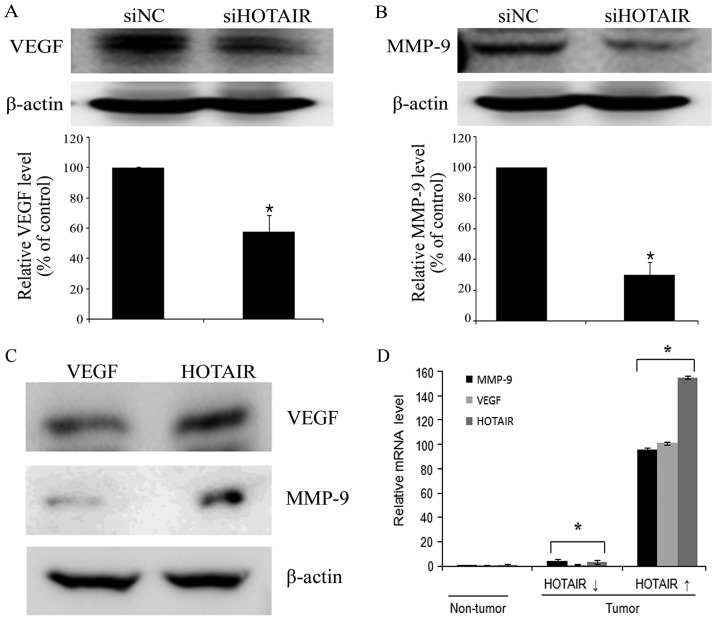
*HOTAIR* increases VEGF and MMP-9 expression in cervical cancer cells. Protein lysates were obtained from siHOTAIR and siNC-transfected HeLa cells 48 h post-transfection. (A) VEGF and (B) MMP-9 expression were analyzed by western blotting. (C) VEGF and MMP-9 levels were analyzed by western blotting in *HOTAIR* overexpression SiHa cells. Band intensities were quantitated, and VEGF and MMP-9 expression were normalized to that of β-actin. (D) VEGF and MMP-9 levels were determined by qRT-PCR in low groups and high *HOTAIR* expression groups of cervical cancer tissues. Each assay was performed in triplicate. Data are mean ± SD. ^*^P<0.05 vs. siNC.

VEGF and MMP-9 play an important role in tumor progression by promoting migration and invasion (28,29). Therefore, the effect of *HOTAIR* on the expression levels of these proteins was determined in HeLa cells. VEGF and MMP-9 protein expressions were significantly lower in siHOTAIR-transfected cells than in siNC-transfected cells (Fig. 4A and B). In contrast, *HOTAIR* overexpression in SiHa cells promoted VEGF and MMP-9 protein expression (Fig. 4C). In addition, the high expression level of *HOTAIR* in cervical cancer tissues associated with upregulation of VEGF and MMP-9 expression levels compared with the low expression groups (Fig. 4D). Taken together, our findings suggest that *HOTAIR* may promote cervical cancer cell migration and invasion through the upregulation of VEGF and MMP-9 expression.

### Inhibition of HOTAIR reversed EMT-related genes in cervical cancer cells

**Figure 5:**
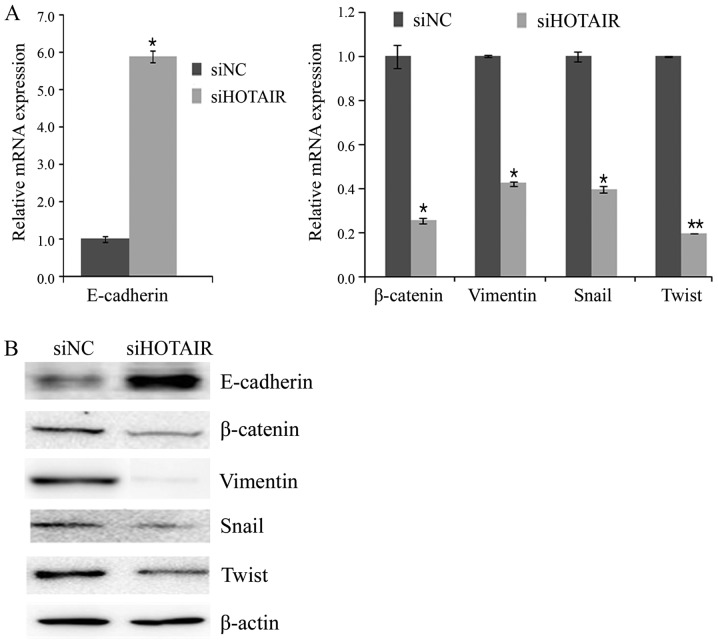
Expression of *HOTAIR* knockdown on the EMT-related genes in HeLa cells. (A) HeLa cells were transfected with *HOTAIR*-specific siRNA and siNC for 48 h. E-cadherin, β-catenin, Vimentin, Snail and Twist expression were analyzed by (A) qRT-PCR and (B) western blotting. Each assay was performed in triplicate. Data are mean ± SD. ^*^P<0.05 vs. siNC, ^**^P<0.001 vs. siNC.

Because the EMT is important in cell migration and invasion, we also investigated whether direct inhibition of *HOTAIR* could reverse EMT-related markers in HeLa cells using real-time RT-PCR and western blot assays following *HOTAIR* knockdown. As anticipated, the siHOTAIR resulted in an increase in the expression of E-cadherin and a decrease in the expression of β-catenin and vimentin (Fig. 5). Next, we assessed the effect of *HOTAIR* knockdown on the expression of following transcription factors known to promote EMT: Snail and Twist. siHOTAIR-transfected cells expressed lower level of snail and twist compared with the siNC-transfected cells (Fig. 5). Collectively, the dysregulation of the expression of EMT-related genes partially explains the involvement of *HOTAIR* in cervical cancer cell migration and invasion.

## Discussion

In the present study, we found that *HOTAIR* expression was higher in cervical cancer tissues than in corresponding non-cancerous tissues and that it was associated with recurrence in cervical cancer patients. Knockdown of *HOTAIR* expression decreased cell growth, migration and invasion in cervical cancer cells. The pro-metastatic effects of *HOTAIR* are likely partially mediated by the regulation of the expression of a number of genes involved in cell migration, invasion and EMT, including VEGF, MMP-9, E-cadherin, β-catenin, Vimentin, Snail and Twist. Together, our findings suggest that *HOTAIR* may represent a potential biomarker and therapeutic target for cervical cancer.

Although the functional role of small regulatory ncRNAs such as miRNAs in human cancers is now well established, little is known about the regulatory roles of lncRNAs and their relevance to human disease. LncRNAs are transcripts of at least 200 nucleotides without protein-coding potential. Like their protein-coding counterparts, many lncRNAs are capped, spliced and polyadenylated (30). Recent data have shown the tissue-specific expression patterns for lncRNAs. Nevertheless, the growing catalog of functionally characterized lncRNAs reveals that these transcripts are important in different physiological processes (31,32), and therefore, altered expression of lncRNAs may promote cancer development and progression (33). Recently, the lncRNA *HOTAIR* was associated with metastatic progression in human breast cancer, hepatocellular carcinoma, cervical and pancreatic cancer (16–19). In the present study, *HOTAIR* expression was associated with disease recurrence in cervical cancer patients and increased the proliferation, migration, and invasion of cervical cancer cells *in vitro*. Recent reports have shown that lncRNAs are crucial for the regulation of chromatin structure, gene expression and translational control (34,35). However, the detailed functional impact and clinical significance of lncRNA-mediated changes in chromatin and gene expression remain to be elucidated. *HOTAIR* recruits PRC2 to specific target genes in the genome, which leads to H3K27 trimethylation and epigenetic silencing of metastatic suppressor genes (16). Therefore, modifications of DNA-binding proteins by *HOTAIR* regulates global gene expression. Kogo *et al* (18) showed that *HOTAIR* expression was closely correlated with PRC2 occupancy in colorectal cancer patients. Furthermore, in a recent study, *HOTAIR*-mediated chromatin changes promoted breast cancer metastasis (16). The fact that *HOTAIR* drives genome-wide chromatin reprogramming suggests that long-range regulation by lncRNAs may be a widespread mechanism. This is supported by a study showing that >20% of tested lncRNAs are bound by PRC2 and other chromatin modifiers (13). These findings provoke questions regarding the initial triggers for *HOTAIR* overexpression and whether understanding of lncRNA mechanics may have clinical relevance.

The recurrence rate after radical surgery in stage I-II cervical cancer is ~15–30%, and the prognosis of recurrent patients is suboptimal (36). Therefore, identification of reliable biomarkers for predicting recurrence is needed to improve the prognosis of cervical cancer patients. Pelvic lymph node metastasis is the most important postoperative risk factor for recurrence or failure to survive, and thus, cervical cancer patients with metastasis in the pelvic lymph nodes require adjuvant therapy (21,37,38). In the present study, we showed that high *HOTAIR* expression was correlated with lymph node metastasis and recurrence in cervical cancer. Therefore, analysis of *HOTAIR* expression in cervical cancer patients may predict the risk of recurrence and, therefore, help guide treatment decisions. Despite the prognostic significance of *HOTAIR* for tumor recurrence, the results of the present study should be viewed cautiously because of the relatively small sample size. Larger prospective studies are needed to confirm our findings.

*HOTAIR* has been shown to increase the invasion of many types of cancer cells including pancreatic, breast, colon, and liver cancer cells (16–18). In the present study, we found that downregulation of *HOTAIR* expression decreased cervical cancer cell proliferation, migration and invasion. Therefore, *HOTAIR* exerts pro-oncogenic activities in cervical cancer and may promote a more aggressive and metastatic phenotype. MMPs play a crucial role in cancer cell invasion and metastasis. MMP-9, which degrades basement membrane collagen, has been shown to promote tumor cell invasion and metastasis and decrease survival in many types of cancer (29,39). It has been generally accepted that tumor angiogenesis plays a critical role in tumor growth, invasion and metastasis. Among the angiogenic factors, VEGF has been shown to have a pivotal role in tumor angiogenesis (40). Knockdown of *HOTAIR* was associated with reduced expression of VEGF and MMP-9 in BEL7402 hepatocellular carcinoma cells (41). Furthermore, *HOTAIR* knockdown inhibited proliferation, migration, and invasion through modulation of the extracellular matrix. We also found that downregulation of *HOTAIR* decreased the expression of VEGF and MMP-9. Taken together, our findings demonstrate that *HOTAIR* accelerates the aggressiveness of cervical cancer cells through the upregulation of VEGF and MMP-9.

The functional importance of *HOTAIR* for the activation of invasion indicates that further studies should identify the role of *HOTAIR* in EMT process (15). It has been demonstrated that knockdown of *HOTAIR* could reverse EMT process in gastric cancer cells (42). These findings prompted us to determine whether *HOTAIR* promotes cervical cancer metastasis by regulating the expression of EMT-related genes. As expected, our data suggest that *HOTAIR* knockdown was dysregulated the expression of EMT-related genes (E-cadherin, β-catenin, Vimentin, Snail and Twist), implying that these genes participate in *HOTAIR*-induced cervical cancer metastasis.

In conclusion, our results suggest that *HOTAIR* is associated with recurrence in cervical cancer. Moreover, *HOTAIR* may promote cervical cancer progression by inducing cell migration and invasion through the upregulation of VEGF, MMP-9 and expression of EMT-related genes. Thus, *HOTAIR* may represent a potential therapeutic target and a prognostic marker for cervical cancer.

## Figures and Tables

**Table I:** Association between *HOTAIR* expression and clinicopathologic factors in cervical cancer (n=111).

		*HOTAIR* expression		
			
	n (%)	Low	High	P-value^a^
Age (mean ± SD)	111	50.4±2.51	50.8±1.29	0.8809
Stage				0.7671
I	43 (38.74)	10	33	
II	56 (50.45)	10	46	
III–IV	12 (10.81)	2	10	
Cell type				0.2334
SCC	78 (70.27)	17	61	
Adeno	24 (21.62)	2	22	
Mixed	3 (2.7)	1	2	
Other	6 (5.41)	2	4	
Tumor size (cm)				0.8839
<4	66 (60)	14	52	
≥4	44 (40)	8	36	
Lymphatic invasion				
Yes	58 (52.25)	10	48	0.6351
No	53 (47.75)	12	41	
Lymph node metastasis				0.0437
Yes	35 (31.53)	3	32	
No	76 (68.47)	19	57	

a Chi-square test or Fisher’s exact test were used to calculate P-values.

Adeno, adenocarcinoma; SCC, squamous cell carcinoma.

**Table II:** Multivariate analysis for recurrence in cervical cancer patients.

	Recurrence		
	
Factor	HR	95% CI	P-value
*HOTAIR* (Low vs. high)	5.281	1.005–27.742	0.0493
Age	0.949	0.907–0.993	0.024
Stage (I vs. II)	0.484	0.148–1.582	0.2298
Stage (I vs. III–IV)	2.428	0.484–12.168	0.2807
Cell type (SCC vs. adeno)	2.288	0.768–6.819	0.1375
Cell type (SCC vs. mixed)	44.548	8.469–234.335	<0.001
Cell type (SCC vs. other)	4.607	0.906–23.411	0.0655
Tumor size (<4 vs. ≥4 cm)	1.651	0.529–5.152	0.3876
Lymphatic invasion (Yes vs. no)	0.974	0.391–2.426	0.9543
Lymph node metastasis (Yes vs. no)	0.824	0.265–2.561	0.7384

Adeno, adenocarcinoma; SCC, squamous cell carcinoma; HR, hazard ratio; CI, confidence interval.

## Abbreviations

lncRNAs: long non-coding RNAs
HOTAIR: Hox transcript antisense intergenic RNA
VEGF: vascular endothelial growth factor
MMP-9: matrix metalloproteinase-9
EMT: epithelial-to-mesenchymal transition
miRNAs: microRNAs
